# Outcomes of a single-step endoscopic ultrasound-guided drainage of pancreatic-fluid collections using an electrocautery-enhanced coaxial lumen-apposing, self-expanding metal stent with and without fluoroscopy

**DOI:** 10.1093/gastro/goaa020

**Published:** 2020-06-04

**Authors:** Babatunde Olaiya, Parit Mekaroonkamol, Bai-Wen Li, Julia Massaad, Cicily T Vachaparambil, Jennifer Xu, Vladamir Lamm, Hui Luo, Shan-Shan Shen, Hui-Min Chen, Steve Keilin, Field F Willingham, Qiang Cai

**Affiliations:** 1 Department of Internal Medicine, Marshfield Clinic, Marshfield, WI, USA; 2 Division of Digestive Diseases, Emory University School of Medicine, Atlanta, GA, USA; 3 Division of Gastroenterology, King Chulalongkorn Memorial Hospital, Chulalongkorn University and Thai Red Cross, Bangkok, Thailand; 4 Department of Gastroenterology, Shanghai General Hospital, Shanghai Jiaotong University, Shanghai, P. R. China; 5 Department of Gastroenterology, Xijing Hospital, Xi’an, Shaanxi, P. R. China; 6 Department of Gastroenterology, Nanjing Drum Tower Hospital, Nanjing, Jiangshu, P. R. China; 7 Department of Gastroenterology, Renji Hospital, Shanghai Jiaotong University, Shanghai, P. R. China

**Keywords:** pancreatic-fluid collections, electrocautery-enhanced coaxial lumen-apposing, self-expanding metal stent (ELAMS), fluoroscopy, endoscopic ultrasound

## Abstract

**Background:**

Fluoroscopy is often used during the endoscopic drainage of pancreatic-fluid collections (PFCs). An electrocautery-enhanced coaxial lumen-apposing, self-expanding metal stent (ELAMS) facilitates a single-step procedure and may avoid the need for fluoroscopy. This study compares the treatment outcomes using ELAMS with and without fluoroscopy.

**Methods:**

Patients with PFCs who had cystogastrostomy from January 2014 to February 2017 were enrolled. Two groups were studied based on fluoroscopy use. Technical success was defined as uneventful insertion of ELAMS at time of procedure. Clinical success was defined as (i) clinical resolution of symptoms after the procedure and (ii) >75% reduction in cyst size on computed tomography 8 weeks after stent placement. Adverse events including bleeding, stent migration, and infection were recorded.

**Results:**

A total of 21 patients (13 males) had PFCs drainage with ELAMS in the study period. The mean age was 51.6 ± 14.2 years. Thirteen patients had walled-off necrosis while eight had a pancreatic pseudocyst. The mean size of the PFCs was 11.3 ± 3.3 cm. Fluoroscopy was used in seven cases (33%) and was associated with a longer procedure time compared to non-fluoroscopy (43.1 ± 10.4 vs 33.3 ± 10.5 min, *P *=* *0.025). This association was independent of the size, location, or type of PFCs. Fluoroscopy had no effect on the technical success rates. In fluoroless procedures, the clinical resolution was 91% as compared to 71% in fluoroscopy procedures (*P *=* *0.52) and the radiologic resolution was 57% as compared to 71% in fluoroscopy procedures (*P *=* *0. 65). Three cases of stent migration/displacement occurred in the fluoroless procedures.

**Conclusions:**

ELAMS may avoid the need for fluoroscopy during cystogastrostomy. Procedures without fluoroscopy were significantly shorter and fluoroscopy use had no impact on the technical or clinical success rates.

## Introduction

Pancreatic-fluid collections (PFCs) are frequently encountered in clinical practice as sequelae of pancreatitis [1, 2]. They are estimated to occur in 5%–16% and 40% of patients with acute and chronic pancreatitis, respectively [3, 4]. While asymptomatic PFCs are often managed conservatively, patients with PFCs who have symptoms including pain, nausea, vomiting, biliary obstruction, or fever often need clinical intervention. While surgical and percutaneous drainage represent viable treatment options, the current treatment paradigm strongly favors minimally invasive therapeutic procedures such as endoscopy over surgical or percutaneous drainage. Surgery remains a very effective method of drainage, although, due to the invasiveness and high morbidity and mortality rates, it is often employed as a backup if percutaneous therapy or endoscopic drainage fails. Percutaneous drainage is less invasive than surgery but carries high risks of fistula formation, bleeding, and infection [5]. Studies comparing clinical outcomes amongst these treatment approaches show that patients who undergo endoscopic drainage have higher treatment success rates, shorter hospital stays, and reduced overall costs as compared to those undergoing percutaneous or surgical drainage [6, 7]. Furthermore, the diagnostic profile of endoscopy has increased significantly with the increasing use of endoscopic ultrasound (EUS).

Transmural drainage of PFCs usually involves the use of endoscopy, EUS, and possibly fluoroscopy in a single- or two-step approach. The two-step procedure involves using radial and linear endoscopy to evaluate the cyst characteristics, identifying a puncture site, introducing a needle to puncture the cyst, introducing a guide wire under ultrasound and/or fluoroscopy guidance, dilating the fistula with a balloon catheter, and inserting a plastic or metal stent. These steps significantly elongate the procedure time, with implications for exposure to anesthesia and radiation from fluoroscopy for patients and medical personnel. A single-step technique utilizes an all-in-one stent-introduction system that significantly reduces the procedure times by eliminating the need to remove and reintroduce needles, wires, dilation catheters, or other accessories.

In comparison, an electrocautery-enhanced coaxial lumen-apposing, self-expandable metallic stent (ELAMS) is an all-in-one stent-introduction system with a preloaded self-expandable metallic (SEM) stent, delivery catheter, and electrocautery system. Its unique design involving a dumbbell configuration and wide diameter decreases the risk of migration, occlusion, and infection—common challenges with plastic and other metallic stents [8–10]. As a single introducer system, ELAMS could lead to even faster procedure times by eliminating the need for a needle and guide wire, as it can be deployed directly without creating an initial fistula. Prior to ELAMS, studies have shown fluoroless EUS-guided drainage of PFCs to be highly efficient and suggested fluoroscopy be used only in collections with a thick wall [11]. However, by enabling direct access to the pancreatic-fluid collection via electrocautery, ELAMS potentially eliminates the need for fluoroscopy, which was previously used for needle and guide-wire placement.

Few studies compare the treatment outcomes with and without fluoroscopy in addition to EUS guidance. In this study, we aim to compare the efficacy and safety of ELAMS stent in the endoscopic drainage of PFC with and without fluoroscopy guidance.

## Materials and methods

### Patient selection and data collection

Institutional review-board approval was obtained and a waiver of informed consent was granted for this Health Insurance Portability and Accountability Act-compliant retrospective study. Clinical databases were searched to identify patients who had ELAMS stent insertion for symptomatic PFCs between 1 January 2014 and 28 February 2017.

A retrospective chart review of the electronic medical records of these patients was done to document patient characteristics at the time of stent insertion, including age, gender, presenting symptoms, and any medical history related to pancreatic dysfunction. Complications secondary to the procedure were also recorded. Pre-procedure and post-procedure cyst sizes were determined by EUS and computed tomography (CT), respectively. PFCs were classified according to the revised 2012 Atlanta Classification. For analytical purposes, the cyst size was recorded as zero where the CT reports read complete resolution or minimal or trace remains of PFC. The length of the procedure and use of fluoroscopy were collected from procedure records. Patients with a prior history of cystogastrostomy or cystoduodenostomy were excluded from the study.

### Endoscopic ELAMS stent insertion

All procedures were performed by an interventional echoendosonographer (Q.C. or F.W.) with a varying degree of trainee involvement in most cases, depending on their stage of training and the endoscopist’s discretion. All procedures were performed under general anesthesia in the endoscopy suite with the patient in either the left lateral or the supine position, depending on the need for fluoroscopic guidance and the endoscopist’s preference. All patients underwent tracheal intubation in a mobile bed. For the group without fluoroscopic guidance, the procedure was performed on the mobile bed. For the group with fluoroscopy guidance, patients had to be moved onto the fluoroscopy bed after intubation. The transferring time (time elapsed on transferring the patient from the mobile bed to the fluoroscopy bed) was also included in the study.

Patients were kept NPO after midnight prior to the procedure. Prophylactic antibiotics were administered using 4.5 grams of piperacillin/tazobactam intravenously or 500 mg of levofloxacin intravenously if the patient was allergic to penicillin shortly before or during the procedure. The antibiotics were continued for 3 days after the procedure.

An esophagogastroduodenoscopy was performed first, before stent placement, using a gastroscope (GIF–H190; Olympus, Tokyo, Japan). The esophagus and the stomach were cleared of any retained particulate matter with water lavage and suction. The routine forward-view examination allowed the endoscopist to evaluate for other possible pathologies and identify any areas with extrinsic compression.

Then, a linear echoendoscope (GF–UCT180; Olympus, Tokyo, Japan) was used for the echoendoscopic portion of the procedures. Under sonographic guidance, the PFC was carefully evaluated for internal vessel, solid debris, and location in relation to the gastric wall. An effacement between the gastric wall and the cystic wall with <1-cm distance and absence of vascular structures in the needle trajectory were confirmed under sonographic guidance before stent placement.

An ELAMS (Boston Scientific, USA) was placed by puncturing into the fluid collection either directly under a ‘pure-cut’ generator setting or over the guide wire after the cyst was punctured by a fine-needle aspiration needle. The inner flange was deployed first under endosonographic guidance. The opened flange was then pulled into the cyst wall, pressing on its opposing stomach wall, and then the outer flange was deployed inside the gastric lumen, confirmed by either endoscopy or fluoroscopy or both. Carbon dioxide was used for insufflation (UCR, Olympus, Japan) in all cases during the entire length of the procedure.

**Figure 1.**
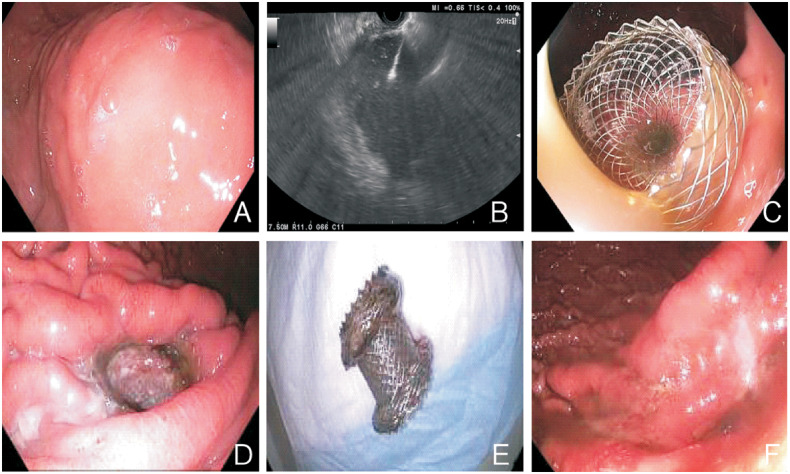
Illustration of steps in the endoscopic ultrasound-guided drainage of pancreatic-fluid collections. (A) Extrinsic compression by pancreatic cyst. (B) Ultrasound view of cystogastrostomy. (C) Stent at insertion. (D) Stent prior to removal. (E) Retrieved stent. (F) Gastrostomy site after stent removal

Puncturing the gastric or duodenal wall, stent deployment, and stent final position were guided or confirmed under EUS with or without additional fluoroscopic guidance. Fluoroscopy was used primarily to monitor the stent-deployment process and evaluate the stent position while other utilities such as evaluation of the needle trajectory while puncturing the fluid collection, aiding in guide-wire placement prior to stent placement, or documenting the stent position for subsequent evaluation when stent migration was suspected were also applied, depending on the endoscopist’s discretion. Cystic fluid was aggressively suctioned to minimize aspiration risk. The procedure is shown in Figure 1 and Video.

After ELAMS placement, the patients were monitored in the hospital for at least one night. Antibiotics were continued for 3 days. If necrosectomy was warranted based on clinical progression and radiologic findings, a repeat upper endoscopy was performed. Endoscopic necrosectomy with or without hydrogen-peroxide lavage was performed as needed. Radiologic imaging was repeated at 4–8 weeks to evaluate the response after cystogastrostomy. If complete resolution was confirmed, then the ELAMS would be removed. If not, repeat endoscopic necrosectomy would be performed as needed.

### Outcomes

Efficacy was measured by the technical and clinical success of the procedure. We defined technical success as the successful insertion of the ELAMS stent at the time of the procedure with visible fluid output. Clinical success was defined as (i) clinical resolution of the presenting symptoms after the procedure and (ii) ≥75% reduction in cyst size (widest diameter) as measured by CT imaging done within 4–8 weeks of the stent insertion.

We defined safety as no adverse events (AEs) due to the procedure at insertion, follow-up, or removal. The AEs include stent migration, bleeding, perforation, infection, and anesthesia-related complications.

### Statistical analysis

Statistical analysis was done with IBM SPSS version 20. Descriptive analysis using frequencies, proportions, measures of central tendency as well as standard deviation were used to answer research questions. Fisher’s exact test was used to assess relationships between categorical variables. Independent sample *t*-test and analysis of covariance (ANCOVA) were utilized for bivariate and multivariate analysis, respectively. The level of significance for all tests was *P *<* *0.05.

## Results

### Patient characteristics

Twenty-one patients (13 males and 8 females) underwent drainage of PFCs using the ELAMS stent in the study period. The mean age at the time of the procedure was 51.6 ± 14.2 years. Thirteen patients (62%) had a walled-off necrosis (WON) while eight (38%) had a pancreatic pseudocyst (PPC). The etiology of the index pancreatitis was indeterminate in six cases (29%). Gallstones were the most commonly identified etiology (29%). Others were alcohol (24%), drug-induced (10%), tobacco (5%), and malignancy (5%). The mean size of the PFC at the time of drainage was 11.3 ± 3.3 cm, five of which were infected (Table 1).

**Table 1.** Summary of patient demographics, pancreatic-fluid-collection characteristics, and treatment outcomes

Variable	Value (*n* = 21)
Age, years, mean ± SD	51.6 ± 14.2
Gender, *n* (%)	
Male	13 (62)
Female	8 (38)
Etiology of pancreatitis, *n* (%)	
Gall stones	6 (29)
Idiopathic/unknown	6 (29)
Alcohol	5 (24)
Drug-induced	2 (10)
Tobacco	1 (5)
Malignancy	1 (5)
Pancreatic-fluid collection, *n* (%)	
Walled-off necrosis	13 (62)
Pseudocyst	8 (38)
Initial cyst size, cm, mean ± SD	11.3 ± 3.3
Infected, *n* (%)	5 (24)
Symptomatic, *n* (%)	19 (90)
Technical success, *n* (%)	21 (100)
Clinical success, *n* (%)	
Clinical resolution	16/19 (84)
Radiological resolution	13 (62)

### Cystogastrostomy with and without fluoroscopy

In all cases, endoscopic access to the pancreatic fluid collection was gained via the stomach (cystogastrostomy). In 90% of the cases (19/21), access to the PFC was gained directly using the electrocautery-enhanced system, followed by the deployment of the SEM stent. In two cases, a needle was used to pierce the gastric wall and gain access to the cyst, and then a guide wire was used to aid deployment of the lumen-apposing stent.

The procedure was completed using a 15 mm (luminal diameter) by 10 mm (saddle length) stent. The mean procedure time was 36.6 ± 9.7 min (range, 17–60 min). During the procedure, fluoroscopy was utilized to assess the correct insertion and positioning of the stent in a third (7/21) of cases. There was a significantly prolonged mean procedure time for fluoroscopy compared to no fluoroscopy guidance (43.1 ± 10.4 vs 33.3 ± 10.5 min, *P *=* *0.025; Table 2). On multivariate analysis using ANCOVA, only fluoroscopy use had a significant effect on the mean procedure time (*P *=* *0.045) while controlling for the type, size, and location of the PFC (Table 3). In addition, it also took a mean time of 6.5 ± 2.3 min to transfer an intubated patient from a mobile bed to the fluoroscopy bed.

**Table 2.** Comparative description of pancreatic-fluid-collection characteristics and treatment outcomes between fluoroscopic and fluoroless procedures

Variable	Fluoroscopy (*n* = 7)	No fluoroscopy (*n* = 14)	*P*-value
Pancreatic-fluid collection, *n* (%)			0.99
Walled-off necrosis	4 (57)	9 (64)	
Pseudocyst	3 (43)	5 (36)	
Initial cyst size, cm, mean ± SD	12.7 ± 2.7	10.7 ± 3.3	0.35
Procedure time, min, mean ± SD	43.1 ± 10.4	33.3 ± 10.5	0.025
Radiologic resolution, *n* (%)	5 (71)	8 (57)	0.65
Clinical resolution, *n* (%)	5 (71)	11/12 (91)	0.52
Adverse events, *n* (%)	0 (0)	3 (21)	0.52

**Table 3.** Analysis of covariance illustrating the relationship between fluoroscopy use, pancreatic-fluid-collection characteristics, and procedure time

Variable	df	Mean square	*F*	*P*-value
Type of collection	1	73.480	0.891	0.36
Size of collection	1	12.714	0.154	0.70
Location of collection	1	12.853	0.156	0.70
Fluoroscopy	1	390.395	4.736	0.045

We recorded a 100% technical success rate. All patients had successful insertion of the stent at the time of the procedure independently of the direct use of the electrocautery system or use of a needle and guide wire to aid SEM stent deployment. Fluoroscopy use also had no effect on the technical success rates, as all stents were placed successfully regardless of fluoroscopy use.

### Follow-up results

Post endoscopic drainage, the clinical resolution of symptoms occurred in 84% (16/19) of the cases. In seven of these, additional intervention, including chemical debridement (*n *=* *5) and balloon sweeping (*n *=* *2) due to stent occlusion, was performed before symptomatic resolution. Clinical symptoms persisted in three patients. One patient developed sepsis and died of septic shock about 5 weeks after the stent insertion. In another patient with a very complex pancreatic history, pain persisted despite prolonged stent-use time (10.4 weeks). In the last patient, stent migration occurred and the stent was removed and subsequently replaced with a pancreatic stent via endoscopic retrograde cholangiopancreatography (ERCP) 2 weeks after the ELAMS insertion. In fluoroless procedures, clinical resolution occurred in 11 of 12 cases (91%) as compared to 5 of 7 cases (71%) in the fluoroscopy procedures. Bivariate analysis did not demonstrate any significant association between fluoroscopy use and clinical resolution of symptoms (*P *=* *0.52; Table 2).

All patients had post-procedure CT follow-up imaging. At 8 weeks post stent insertion, 62% of patients (eight WON, five PPC) demonstrated >75% reduction in cyst size, with complete resolution in 52% of patients. Among the 13 patients with radiologic complete resolution of PFCs, 4 (31%) were lost to follow-up. Of the remaining nine (69%) patients, following an average follow-up time of 14.3 weeks (range, 0.2–36.4 weeks) after stent removal, only one patient had a recurrence of PFC. In the fluoroless procedures, radiologic resolution occurred in 8 of 14 cases (57%) as compared to 5 of 7 cases (71%) in the fluoroscopy procedures. Bivariate analysis did not demonstrate any significant association between fluoroscopy use and radiologic resolution of symptoms (*P *=* *0.65; Table 2).

The stent was removed in 19 patients after an average of 9.3 ± 6.6 weeks. One patient spontaneously passed the stent without any complication by the time of radiologic evaluation at 6 weeks. This was also confirmed by endoscopic evaluation. Further radiologic evaluation 2 weeks later showed partial resolution of the pancreatic pseudocyst. The patient, however, remained asymptomatic. In the other case, the patient was readmitted with features of severe inflammatory response syndrome approximately 3 weeks after stent insertion. The patient was placed on antibiotics but had no surgical interventions due to her poor clinical status and co-morbid conditions. During admission, an attempted necrosectomy was cancelled due to severe hypotension. Despite adequate supportive measures, the patient deteriorated clinically and died of septic shock 2 weeks after readmission.

### AEs

Stent migration was recorded in two patients (10%). Despite stent migration, radiologic and clinical resolution had occurred in one case at the time of stent removal. In the other case, the stent was removed and replaced with 7 Fr x 8-cm plastic stents via endoscopic retrograde cholangiopancreatography (ERCP). Stent dislodgment occurred in one patient (5%), who passed the stent spontaneously as previously described. No other major AEs were recorded. All cases of stent migration/displacement occurred in patients who had fluoroless procedures. Regardless, bivariate analysis did not demonstrate any significant association between fluoroscopy use and the risk of AEs, i.e. stent migration/displacement (*P *=* *0.52; Table 2).

## Discussion

The potential harmful effects of radiation and improved endoscopic imaging and techniques have led to reduced dependence on and use of fluoroscopy in the evaluation and treatment of gastrointestinal pathologies [12]. This is especially evident with the novel ELAMS that reduces the need for fluoroscopy in the management of PFCs by allowing a single-step stent-deployment procedure. Anderloni *et al.* recently described a new successful technique involving intra-channel stent release under EUS guidance that eliminates the need for either endoscopic or fluoroscopic views altogether [13]. Our study compares the efficacy of ELAMS insertion using fluoroscopy vs non-fluoroscopy. ELAMS insertion without fluoroscopy omits the need for transferring patients from a mobile bed to the fluoroscopy bed, reduces the cystogastrostomy time, and limits unnecessary patient exposure to radiation.

For our study, we observe a 100% technical success rate, which is consistent with the high technical success rates reported in the literature [14–17]. We also observe that fluoroscopy use had no effect on the technical success rates. All stents were placed successfully whether or not fluoroscopy was utilized. Similarly, we observed no significant differences in the clinical success rates either by clinical or radiologic criteria between the two groups. Furthermore, we observe difference success rates defined by clinical (84%) and radiologic (62%) criteria. Although not statistically significant in our study, this may suggest that, despite a suboptimal reduction in the cyst size, some patients still achieved clinical resolution of symptoms and this could impact future clinical practice. As recommended in the revised Atlanta classification, initial clinical intervention in PFCs should be guided by clinical symptoms alone and not the cyst size, as opposed to the original criteria [18]. Similarly, clinical symptoms rather than radiologic imaging should guide endoscopists for further intervention after stent placement.

The most interesting finding of the study was that EUS-guided endoscopic cystogastrostomy by using the ELAMS was more efficient when performed without fluoroscopy. Transfer of an intubated patient from a mobile bed to the fluoroscopy bed took up to 10 min or longer. In addition, the average procedure time was significantly shorter (10 min per procedure) than that with fluoroscopy. This association was independent of the size or location of the PFC or whether the PFC was a pseudocyst or a WON. Furthermore, the technical and clinical success rates remained similar between the two groups.

Our study showed that all three cases of stent migration or displacement occurred in patients who had fluoroless procedures. However, this finding was not statistically significant. As such, it is unlikely that fluoroless procedures increase the risk of stent migration or displacement. Previous reports have demonstrated the benefits of fluoroscopy in the repositioning or removal of displaced stents during endoscopic drainage [19, 20], although there are no clear benefits of fluoroscopy during stent insertion while using ELAMS. Furthermore, the 2D view of fluoroscopy is inferior to EUS in assessing whether stent migration vertical to the X-ray has occurred during stent insertion [21]. Though stent dislodgment can have potential serious outcomes, the patient in our study made an uneventful recovery with full resolution of the PFC. A follow-up CT scan did not locate the stent, raising the high probability of spontaneous stent passage per rectum.

Our study has several limitations. It is a single-institution experience with inherent patient-selection bias. Beyond its retrospective nature, the study sample is small and, as such, may be underpowered. Furthermore, we observed three AEs (stent migration/displacement) in patients who had fluoroless procedures. This observation emphasizes the need for larger studies to further evaluate the risk of AEs in fluoroless procedures. Outcomes in our study may also be performer-dependent and there were only two interventional echoendoscopists accompanied by trainees performing ELAMS in our study. Lastly, we employed two different imaging techniques, i.e. EUS and CT imaging, to measure the PFC size pre and post stent insertion, respectively, potentially causing measurement bias.

In conclusion, ELAMS may avoid the need for fluoroscopy during cystogastrostomy. Procedures without fluoroscopy were significantly shorter. This approach also reduces the lab load of the staff by omitting transferring patients from a mobile bed to a fluoroscopy bed. All patients had successful stent insertion and more than four-fifths (84%) of the patients had successful resolution of symptoms, indicating a benefit of the procedure. While AEs occurred in 3 of 21 cases, only one patient needed surgical intervention due to stent migration. We conclude that ELAMS without fluoroscopy is a safe and efficient therapeutic option in the management of PFCs. Further larger-scale studies and multicenter studies are needed to validate this finding.

## Author’s contributions

B.O., B.W.L. and P.M. collected and analysed the data, performed statistical work, and drafted and revised the paper. C.T.V., J.X., V.L., H.L., J.M., S.S.S., H.M.C. and S.K. participated in discussion and critically reviewed and revised the paper. F.F.W. performed the procedure and critically reviewed and revised the paper. Q.C. performed the procedure, conceptualized and designed the study, and critically revised all the intellectual content of the manuscript. All authors read and approved the final version of the manuscript.
